# Computational and Pharmacological Evaluation of Carveol for Antidiabetic Potential

**DOI:** 10.3389/fphar.2020.00919

**Published:** 2020-07-29

**Authors:** Muhammad Shabir Ahmed, Arif-ullah Khan, Lina Tariq Al Kury, Fawad Ali Shah

**Affiliations:** ^1^ Riphah Institute of Pharmaceutical Sciences, Riphah International University, Islamabad, Pakistan; ^2^ College of Natural and Health Sciences, Zayed University, Abu Dhabi, United Arab Emirates

**Keywords:** carveol, molecular docking, alpha-amylase, antidiabetic, anti-hyperlipidemic, hepatoprotective

## Abstract

**Background:**

Carveol is a natural drug product present in the essential oils of orange peel, dill, and caraway seeds. The seed oil of *Carum Carvi* has been reported to be antioxidant, anti-inflammatory, anti-hyperlipidemic, antidiabetic, and hepatoprotective.

**Methods:**

The antidiabetic potential of carveol was investigated by employing *in-vitro, in-vivo*, and *in-silico* approaches. Moreover, alpha-amylase inhibitory assay and an alloxan-induced diabetes model were used for *in-vitro* and *in-vivo* analysis, respectively.

**Results:**

Carveol showed its maximum energy values (≥ -7 Kcal/mol) against sodium-glucose co-transporter, aldose reductase, and sucrose-isomaltase intestinal, whereas it exhibited intermediate energy values (≥ -6 Kcal/mol) against C-alpha glucosidase, glycogen synthase kinases-3β, fructose-1,6-bisphosphatase, phosphoenolpyruvate carboxykinase, and other targets according to *in-silico* analysis. Similarly, carveol showed lower energy values (≥ 6.4 Kcal/mol) against phosphoenolpyruvate carboxykinase and glycogen synthase kinase-3β. The *in-vitro* assay demonstrated that carveol inhibits alpha-amylase activity concentration-dependently. Carveol attenuated the *in-vivo* alloxan-induced (1055.8 µMol/Kg) blood glucose level in a dose- and time-dependent manner (days 1, 3, 6, 9, and 12), compared to the diabetic control group, and further, these results are comparable with the metformin positive control group. Carveol at 394.1 µMol/Kg improved oral glucose tolerance overload in rats compared to the hyperglycemic diabetic control group. Moreover, carveol also attenuated the glycosylated hemoglobin level along with mediating anti-hyperlipidemic and hepatoprotective effects in alloxan-induced diabetic animals.

**Conclusions:**

This study reveals that carveol exhibited binding affinity against different targets involved in diabetes and has antidiabetic, anti-hyperlipidemic, and hepatoprotective actions.

## Introduction

Diabetes mellitus (DM) is a leading health issue, having a highly troubling frequency in developing countries. The primary risk factors of DM include a sedentary lifestyle, poor nutritional habits, and obesity (Hu, 2011). Diabetes is an independent risk factor and important comorbidity of several human diseases and increases the risk of death by 1.5–3 times (Capes et al., 2001). The WHO ranked DM the biggest epidemic and the most common cause of functional disabilities (Mathers and Loncar, 2006; Shah et al., 2019). DM is predominantly related to increased blood glucose level associated with alteration in the breakdown of fats, proteins, and carbohydrates due to either a decrease in the production of insulin. The low insulin level in type I diabetes is due to autoimmunity in beta cells of the pancreas (Tuomi, 2005), and the resulting hyperglycemia can be managed by administering insulin injections subcutaneously. In type II diabetes, there is rather a reduction in insulin sensitivity in hepatic, cardiac, and fat cells, and this can be managed by hypoglycemic drugs (Nathan et al., 2009). The foremost risk factors for the progression of anomalous secretion of insulin or resistance to it include genetic defects, aging, viral infection, environmental factors, and a sedentary lifestyle combined with high calorie intake (Saltiel, 2001).

Type II DM has long-term health hazards, including neuropathy, nephropathy, and accelerated atherosclerosis leading to an increased risk of myocardial infarction (MI) (Hanefeld et al., 1996; Viberti, 2005). Furthermore, it makes the human body prone to dyslipidemia, high blood pressure, and obesity (Association, 2014). Moreover, dyslipidemia triggers various cardiovascular complications such as atherosclerosis, MI, hypertension, and obesity-related problems (Ashfaq et al., 2017). Stress also plays an important role in the progression of hyperlipidemia due to the generation of free radicals, which may lead to atherosclerosis and cardiovascular diseases (Arise et al., 2016).

Alloxan is a potent inducer of pancreas toxicity and is therefore used for experimental diabetes induction (Brenna et al., 2003). Alloxan induces a multiphasic blood glucose response when injected into an experimental animal, with consistent fluctuations in the plasma insulin level associated with structural damage to beta cells (Olusanya and Ifeoluwa, 2018). The pathology of DM is attenuated by insulin therapy and orally by hypoglycemic medications such as biguanides and sulfonylureas. However, the therapeutic potential of these oral synthetic antidiabetic agents has been limited by long-term microvascular and macrovascular complications (Stenman et al., 1990; Spiller and Sawyer, 2006).

**Figure 1:**
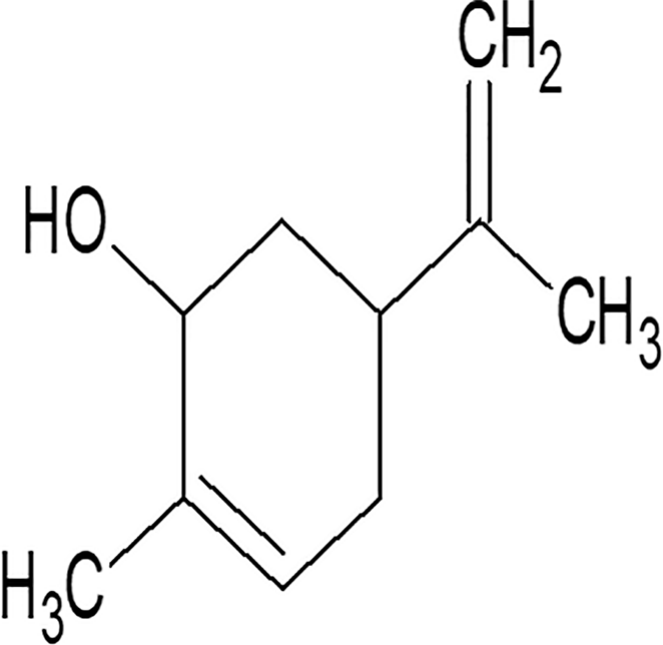
Chemical structure of carveol.

Carveol (**Figure 1**) is a natural drug substance present in the essential oils of orange peel, dill (*Anethum graveolens* L.), and caraway seeds (Crowell et al., 1992). The chemical components are D-limonene, mono-terpenes carveol acetate, and trans- and cis-carveol, glyceryl esters (Singh and Lal, 2008). The seeds of caraway oil (Carum carvi L.) have been described in customary Chinese medicine as antispasmodic, carminative, astringent, anti-inflammatory, anti-cancer, and hepatoprotective (Johri, 2011; Agrahari and Singh, 2014).

This study aims to demonstrate the antidiabetic potential of carveol through molecular docking, *in-vitro* study, and an *in-vivo* animal experimentation model using alloxan-induced diabetes and to further evaluate the anti-hyperlipidemic and hepatoprotective effect of carveol.

## Materials and Methods

### Chemicals

Alloxan monohydrate (CAS number 22-44-11-3), carveol (99-48-9), acarbose (56180-94-0), alpha-amylase (A6255), and metformin hydrochloride (11-15-70-4) were acquired from Sigma-Aldrich Co. LLC, USA. All chemicals were analytical grade (99% HPLC grade).

### Animals

Adult Sprague–Dawley (SD) rats of either sex weighing 250–280 g and 7–11 weeks old were obtained from the local breeding facility of Riphah International University. The animals were kept in a standard animal room temperature at 18–22°C under circadian light and dark conditions with access to food and water ad libitum. All the experimental protocols were recommended and approved by the Research and Ethical Committee (REC) of the Riphah Institute of Pharmaceutical Sciences (RIPS) (Approval ID: Ref. No.: REC/RIPS/2019/03).

### Computational Studies

The standard drugs used for *in-silico* analysis were miglitol, metformin, carbenoxolone, thiadiazolidinone-8 (TDZD-8), rosiglitazone, acarbose, and sergliflozin. 3D structures of human protein targets involved in DM were obtained from the online Protein Data Bank (PDB) (www.rcsb.org) (Sussman et al., 1998). The target proteins selected were: alpha-amylase (AA, PDB ID: 3BA1), C-alpha glucosidase (C-AG, PDB ID: 3L4T), aldose reductase (AR, PDB ID: 2R24), phosphoenolpyruvate carboxykinase (PEPCK, PDB ID: 1NI4), fructose-1,6-bisphosphatase (FBP1, PDB ID: 5ZWK), 11β-hydroxysteroid dehydrogenase-1 (11β-HSD1, PDB ID: 3D3E), glycogen synthase kinase-3β (GSK-3β, PDB ID: 6GJO), peroxisome proliferator-activated receptor-γ (PPAR-γ, PDB ID: 4EMA), phosphatidylinositol 3 kinase (PI3K, PDB ID: 1E90), sucrase-isomaltase intestinal (SIMI, PDB ID: 3TOP), and sodium-glucose co-transporter (SGLT, PDB ID: 2XQ2). By using discovery studio visualizer (DSV), water molecules and ligands were removed, and polar H-atoms were added, and the resulting structure was saved in PDB format. Molecular docking was performed by the Auto Dock tool, v.1.5.6, and the PyRx 0.8 docking tool (Duhovny et al., 2002). Docking was executed and evaluated on the basis of atomic contact energy (ACE) value (Kcal/mol). The best poses were evaluated, and the one with the lowest ACE value (Kcal/mol) was selected for evaluation through Biovia DSV. Each complex was assessed in a 3D pattern to check the maximum binding interactions formed between ligands and amino acid residues of the protein targets.

### Alpha-Amylase Inhibitory Assay

The antidiabetic potential of the test compound carveol was determined by α-amylase inhibition assay following the standard protocol with minor modification (Kim et al., 2000). The reaction mixture containing 15 μl phosphate buffer (pH 6.8), 25 μl α-amylase enzyme (0.14 U/mL), different concentrations of the sample in normal saline, and 40 μl starch solution (2 mg/mL in potassium phosphate buffer) was incubated at 50°C for 30 min in a 96-well plate followed by addition of 20 μl of 1 M HCl to stop the reaction. Afterward, 90 μl of iodine reagent (5 mM iodine, 5 mM potassium iodide) was added to each well. Negative control was prepared without the sample, whereas blank was prepared without the sample and amylase enzyme, each being replaced by equal quantities of the buffer. Acarbose (250 μM/Kg) was used as a positive control. The absorbance of the reaction plate after incubation was measured at 540 nm. The activity was expressed as percent α-amylase inhibition and calculated by the following equation:

%α−amylase inhibition = (Os−On)/(Ob−On)×100

where On = Absorbance of negative control, Os = Absorbance of sample, and Ob = Absorbance of the blank well.

### Blood Glucose Levels and Body Weight Measurement

SD rats were adjusted to the laboratory environments and reserved for whole night fasting (12–14 h). The animals were divided into six groups, each containing five animals (n=5). Group I and II were non-diabetic control and diabetic control groups injected with saline (10 mL/Kg) and alloxan monohydrate (1055.8 µMol/Kg), respectively. Groups III, IV, and V were alloxan-induced diabetic rats administered with the test compound at doses of 65.7, 197, and 394.1 µMol/Kg respectively. Group VI was positive control and was injected with metformin (1207.5 µMol/Kg). The blood glucose levels were measured at days 1, 3, 6, 9, and 12, using an Accu-Chek instant glucometer. For the complete treatment period, the body weight of animals was measured at the same regular intervals. For the induction of diabetes, alloxan monohydrate was used (Dunn and McLetchie, 1943). Freshly prepared alloxan solution (1055.8 µMol/Kg) in saline was given to experimental rats *via* an intra-peritoneal route (Olusanya and Ifeoluwa, 2018). After 48 h, blood glucose levels of experimental rats were measured by using the tail prick methodology. Rats with blood glucose levels ≥ 200 mg/dL were demarcated as hyperglycemic (Saudek et al., 2008).

### Oral Glucose Tolerance Test (OGTT)

After 18 h of fasting, rats were placed into four groups. Group I and II were non-diabetic and diabetic control and were injected with saline (10 mL/Kg) and alloxan (1055.8 µMol/Kg), respectively. Group III was a carveol-treated (394.1 µMol/Kg) group. Group IV was positive control and injected with metformin (1207.5 µMol/Kg). Each group was pre-treated, and after 30 min, the D-glucose load (3 g/Kg) was administered orally. The blood glucose level was measured at 0, 30, 60, 90, and 120 min using an Accu-Chek instant glucometer (Rubino and Marescaux, 2004).

### Glycosylated Hemoglobin (HbA1C) Test

After 6 weeks of treatment, the HbA1C test was performed for all groups (Asgary et al., 2008). A cardiac puncture methodology was utilized to collect blood samples (Doeing et al., 2003). The HbA1C level was measured in a local laboratory at Islamabad, Pakistan.

### Serum Biomarker Analysis

A hepatic functional test was performed for each group (n=5/group). The cardiac puncture method was used to collect blood samples. The lipid profile in terms of TC, TGs, LDL, and HDL and the hepatic functional markers alanine transaminase (ALT), aspartate aminotransferase (AST), alkaline phosphatase (ALP), and total bilirubin (TB) were analyzed in a local laboratory at Islamabad, Pakistan.

### Statistical Analysis

Data are presented as mean ± standard error of the mean (SEM). The significance of results was evaluated by analysis of variance (ANOVA), followed by a multiple comparison test. *p* < 0.05 was considered to be statistically significant. Statistical assessment, preparation of graphs, and evaluation were performed by using Graph Pad Prism 6.

## Results

### *In-Silico* Analysis

**Figure 2:**
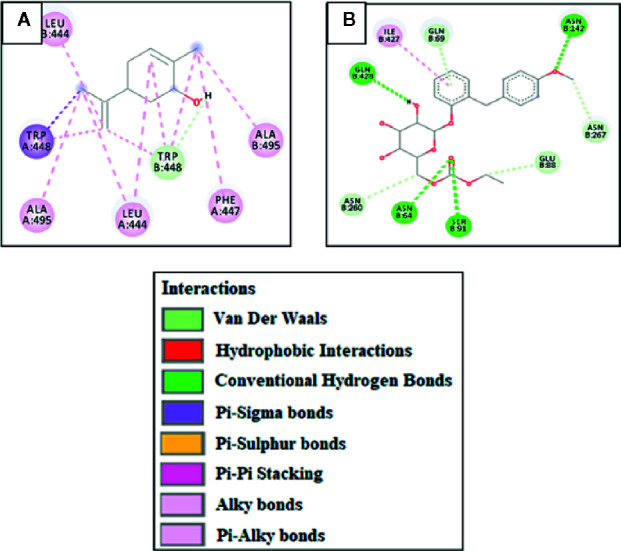
**(A)** and **(B)** represent 2D-interactions of carveol and sergliflozin with sodium-glucose co-transporter (SGLT), respectively, evaluated through Biovia Discovery Studio 2016.

Against AA, carveol and miglitol showed ACE values of -6.2 and -6.3 Kcal/mol and formed 1 hydrogen bond (H-bond) with ASP197 and 5 H-bonds with ILE312, ASN301, ASP317, and THR314, respectively. Carveol showed hydrophobic interactions with LEU165, HIS299, TRP58, TYR62, and miglitol showed no hydrophobic interactions. Against C-AG, carveol and miglitol showed ACE values of -6.5 and -5.8 Kcal/mol and showed no H-bonds against C-AG and 3 H-bonds with LYS1099, SER1012, and TYR1044, respectively. Carveol showed hydrophobic interactions with HIS1584, TRP1355, TRP1418, TYR1251, PHE1559, and ILE1315, and miglitol showed no hydrophobic interactions. against AR, carveol and metformin exhibited ACE values of -7.1 and -5.3 Kcal/mol and made no H-bonds and 2 H-bonds with SER210 and GLN183, respectively. Carveol showed hydrophobic interactions with TRP20, TRP111, TYR209, SER210, HIS110, and ILE260, and metformin formed hydrophobic interactions with ASP43 and TYR209. Against PEPCK, carveol and metformin exhibited ACE values of -6.5 and -4.7 Kcal/mol and formed no H-bonds and 3 H-bonds with ARG438, GLN112, and GLU89, respectively. Carveol showed hydrophobic interactions with PHE517, PHE525, PHE530, and TRP516, and metformin showed hydrophobic interactions with GLU89. Against FBP1, carveol and metformin showed ACE values of -6.6 and -5.1 Kcal/mol and formed 3 H-bonds with THR171, SER45, and ARG49 and 2 H-bonds with ASP118 and LEU120, respectively. Carveol showed hydrophobic interactions with PRO188, ARG49, and LEU186, and metformin showed hydrophobic interactions with GLU97, GLU98, ASP118, and ASP121. Against 11β-HSD1, carveol and carbenoxolone showed ACE values of -6.6 and -11.2 Kcal/mol and formed 1 H-bond with SER125 and no H-bonds, respectively. Carveol showed hydrophobic interaction with PHE129, HIS135, ASN127, and ALA182 and carbenoxolone with ILE46, ILE121, ALA 223, LEU 217, TYR 177, and TYR183. Against GSK-3β, carveol and TDZD-8 exhibited ACE values of -6.4 and -6.6 Kcal/mol and formed 1 H-bond with ASP200 and 2 H-bonds with LEU88 and GLN89, respectively. Carveol showed hydrophobic interactions with VAL70, VAL110, LEU132, LEU188, ILE62, ALA83, TYR134, and CYS199 and TDZD-8 with PHE67 and VAL87. Against PPAR-γ, carveol and rosiglitazone showed ACE values of -6 and -8.5 Kcal/mol and made 1 H-bond with GLU295 and 4 H-bonds with LEU228, ARG288, and SER289, respectively. Carveol showed hydrophobic interactions with LEU330, LEU333, MET329, ILE326, ARG288, and ALA292 and rosiglitazone with PHE282, CYS285, ALA292, ILE326, LEU330, and HIS449. Against PI3K, carveol and rosiglitazone exhibited ACE values of -6.7 and -7.9 Kcal/mol and formed 1 H-bond with ASP278 and 4 H-bonds with PRO563, PHE497, and THR1043, respectively. Carveol showed hydrophobic interactions with HIS199, HIS693, LYS689, ARG690, MET728, and PRO789 and rosiglitazone with PRO590, LYS591, and ILE1048. Against SIMI, carveol and acarbose exhibited ACE values of -7 and -8.1 Kcal/mol and formed 1 H-bond with GLU1543 and 9 H-bonds with GLN1254, TYR1251, GLN1286, ARG1377, LEU1367, and GLN1372, respectively. Carveol showed hydrophobic interactions with PRO1160, LYS1536, PHE1544, LEU1524, and ALA1554 and acarbose with ILE1587. Against SGLT, carveol and sergliflozin presented ACE values of -7.3 and -9 Kcal/mol and made no H-bonds and 4 H-bonds with SER91, ASN64, ASN142, and GLN428, respectively. Carveol showed hydrophobic interactions with TRP448, LEU444, ALA495, and PHE447 and sergliflozin showed hydrophobic interactions with ILE427 (**Figure 2**). The ACE (Kcal/mol) values for ligand–complex interactions, the targets, the number of H-bonds, the amino acids employed in forming hydrophobic interactions, and the H-bonds of ligand complexes with targeted protein are presented in **Table 1** and **Figures S1–S10**.

**Table 1:** Atomic contact energy (ACE) values (Kcal/mol) of best-docked poses of carveol and standard drugs, as well as hydrogen bonds and hydrophobic interactions formed, against AA, alpha-amylase; C-AG, C-alpha Glucosidase; AR, aldose reductase; PEPCK, phosphoenolpyruvate carboxykinase; FBP1, fructose-1,6-bisphosphatase; 11b-HSD1, 11b-hydroxysteroid dehydrogenase-1; GSK-3b, glycogen synthase kinases-3b; PPAR-g, peroxisome proliferator-activated receptor-g; PI3K, phosphatidylinositol 3 kinases; SIMI, sucrase-isomaltase intestinal; SGLT, sodium-glucose co-transporter.

		Carveol				Standard Drugs				
Target Proteins	PDB ID	Binding energies (Kcal/mol)	H – bonds	Binding Residue	Hydrophobic interactions	Name	Bindingenergies(Kcal/mol)	H - bonds	Binding Residue	Hydrophobic interactions
AA	3BA1	-6.2	1	ASP 197	LEU 165, TYR 62, TRP 58, HIS 299	Miglitol	-6.3	5	ASP 317, ILE 312, ASN 301, THR 314 (2)	–
C-AG	3L4T	-6.5	–	–	HIS 1584, TRP 1355,1418, TYR 1251, PHE 1559, ILE 1315	Miglitol	-5.8	3	LYS 1099, SER 1012, TYR 1044	–
AR	2R24	-7.1	–	–	TRP 20,111, TYR 209, SER 210, HIS 110, ILE 260, CYS 298	Metformin	-5.3	2	SER 210, GLN 183	ASP 43, TYR 209
PEPCK	1NI4	-6.5	–	–	PHE 517,530,525, TRP 516	Metformin	-4.7	3	ARG 483, GLN 112, GLU 89	GLU 89
FBP1	5ZWK	-6.6	3	THR 171, SER 45, ARG 49	PRO 188(2), ARG 49, LEU186	Metformin	-5.1	2	LEU 120, ASP 118	GLU 97,98, ASP 118,121
11β-HSD1	3D3E	-6.6	1	SER 125	PHE 129, HIS 135, ASN 127, ALA 182	Carbenoxolone	-11.2	–	–	ILE 46,121, ALA 223, LEU 217, TYR 177,183
GSK-3β	6GJO	-6.4	1	ASP 200	VAL 70,110, LEU 132,188, ILE 62, ALA 83, TYR 134, CYS 199	Thiazolidinone	-6.6	2	LEU 88, GLN 89	PHE 67, VAL 87
PPAR-γ	4EMA	-6	1	GLU 295	LEU 330,333, MET 329, ILE 326, ARG 288, ALA 292	Rosiglitazone	-8.5	4	LEU 228, ARG 288, SER 289 (2)	PHE 282, CYS 285, ALA 292, ILE 326, LEU 330, HIS 449
PI3K	1E90	-6.7	1	ASP 278	HIS 199,693, LYS 689, ARG 690, MET 728, PRO 789	Rosiglitazone	-7.9	4	PRO 563, PHE 497, THR 1043 (2)	PRO 590, LYS 591, ILE 1048
SIMI	3TOP	-7	1	GLU 1543	PRO 1160, LYS 1536, PHE 1544, LEU 1524, ALA 1554	Acarbose	-8.1	9	GLN 1254, TYR 1251, GLN 1286 (2) ARG 1377 (2), LEU 1367, GLN 1372 (2)	ILE 1587
SGLT	2XQ2	-7.3	–	–	TRP 448(2), LEU 444(2), ALA 495(2), PHE 447	Sergliflozin	-9	4	ASN 64, ASN142, SER 91, GLN 428	ILE 427

Standard inhibitors or activators of pathways are Miglitol, metformin, carbenoxolone, thiadiazolidinone-8, rosiglitazone, acarbose, and sergliflozin. Amino acids are: alanine (ALA), arginine (ARG), asparagine (ASN), aspartic acid (ASP), cysteine (CYS), glutamine (GLN), glutamic acid (GLU), glycine (GLY), histidine (HIS), isoleucine (ILE), lysine (LYS), methionine (MET), phenylalanine (PHE), proline (PRO), serine (SER), threonine (THR), tryptophan (TRP), tyrosine (TYR), and valine (VAL).

### α-Amylase Inhibition

Carveol and acarbose caused α-amylase enzyme inhibition at different concentrations; the results are shown in **Table 2**.

**Table 2:** Alpha-amylase inhibitory effect of carveol and acarbose.

Carveol		Acarbose	
Concentration(µMol)	% Inhibition(Mean ± SEM)	Concentration(µMol)	% Inhibition(Mean ± SEM)
0.821	14.25 ± 0.01^***^	0.193	28.03 ± 0.003
1.642	23.25 ± 0.005^***^	0.387	39.35 ± 0.008
3.284	29.49 ± 0.002^***^	0.774	59.77 ± 0.006
6.569	41.14 ± 0.004^***^	1.548	71.74 ± 0.003
19.707	50.86 ± 0.004^***^	4.646	78.51 ± 0.002
32.845	61.96 ± 0.003^***^	7.744	88.42 ± 0.003
65.690	73.01 ± 0.002^***^	15.489	98.95 ± 0.002

Values expressed as percentage inhibition mean ± SEM. One-way ANOVA was used for statistical analysis, ^***^p < 0.001 compares the percentage inhibition of carveol group vs. the acarbose group.

### Effect on Blood Glucose Levels

**Figure 3:**
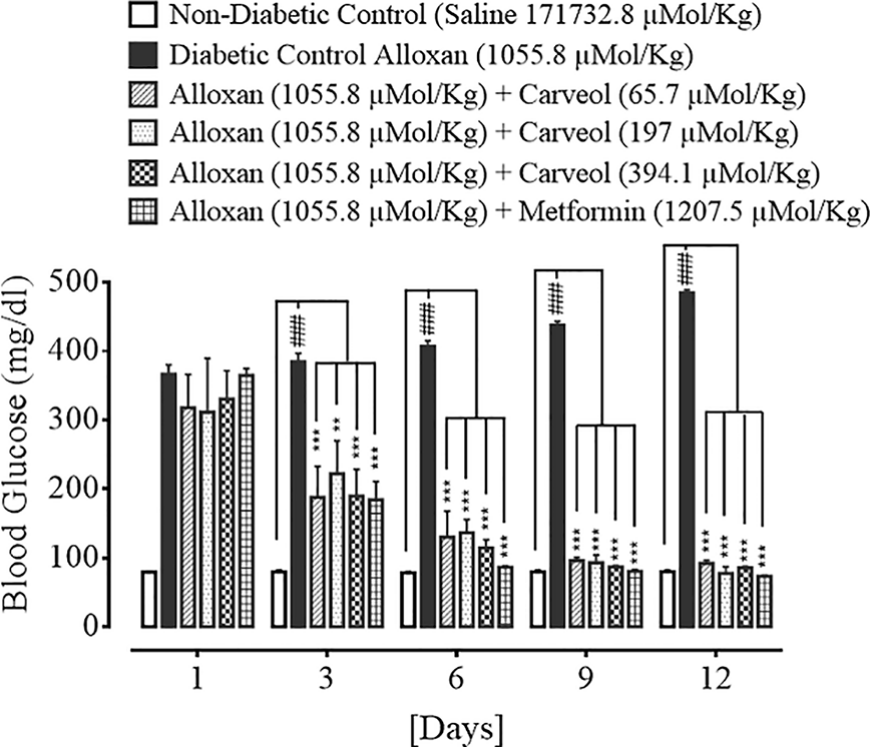
Bar graph representing blood glucose level on different treatment days of the saline-treated group (non-diabetic control), alloxan-treated group (diabetic control), carveol-treated groups at different doses (65.7, 197, 394.1 µMol/Kg), and metformin-treated group against alloxan-induced hyperglycemia in rats. Data presented as mean ± SEM. Statistical analysis used one-way ANOVA, followed by post-hoc Tukey’s test. ^###^ *p* < 0.001 vs. saline group, ^**^ *p* < 0.01 and ^***^ *p* < 0.001 comparison of the blood glucose levels of carveol- and metformin-treated groups vs. diabetic control group.

The levels of blood glucose for the non-diabetic control (saline, 10 mL/Kg), diabetic control alloxan-treated (1055.8 µMol/Kg), carveol-treated (65.7, 197, and 394.1 µMol/Kg), and metformin-treated (positive control) (1207.5 µMol/Kg) groups are shown in **Figure 3**.

### Effect on Body Weight

The body weights of non-diabetic control (saline, 10 mL/Kg), diabetic control alloxan-treated (1055.8 µMol/Kg), carveol-treated (65.7, 197, and 394.1 µMol/Kg), and metformin-treated (positive control) (1207.5 µMol/Kg) groups are shown in **Table 3**.

**Table 3:** Effect of carveol and metformin on different treatment days on body weight (g) of alloxan-induced diabetic rats.

Treatment	Day 1	Day 3	Day 6	Day 9	Day 12
Non-Diabetic Control (Saline, 10 mL/Kg)	205.3 ± 2.45	209.8 ± 4.52	212.6 ± 2.06	216.2 ± 4.12	221.3 ± 3.32
Diabetic Control (Alloxan 1055.8 µMol/Kg)	199.2 ± 9.57	190.5 ± 10.1^##^	185.2 ± 9.58^###^	179.2 ± 9.33^###^	173.7 ± 8.99^###^
Alloxan (1055.8 µMol/Kg)) + Carveol (65.7 µMol/Kg)	172.1 ± 6.31^**^	166.9 ± 5.56^**^	165.2 ± 3.84^**^	164.4 ± 4.13^**^	163.2 ± 4.23^**^
Alloxan (1055.8 µMol/Kg)) + Carveol (197 µMol/Kg)	155.0 ± 8.83^***^	154.8 ± 8.13^***^	154.3 ± 7.60^***^	152.2 ± 8.82^***^	150.6 ± 9.45^***^
Alloxan (1055.8 µMol/Kg)) + Carveol (394.1 µMol/Kg)	162.8 ± 9.19^***^	156.2 ± 8.69^***^	151.4 ± 7.16^***^	150.1 ± 6.78^***^	148.6 ± 6.98^***^
Alloxan (1055.8 µMol/Kg)) + Metformin (1207.5 µMol/Kg)	165.3 ± 6.64^**^	154.4 ± 4.74^***^	149.8 ± 4.64^***^	145.6 ± 4.02^***^	142.6 ± 3.48^***^

Values expressed as mean ± SEM. One-way ANOVA was used for statistical analysis, followed by Bonferroni’s multiple comparisons test. ^##^p < 0.01, ^###^p < 0.001 vs. saline group, ^**^p < 0.01, ^***^p < 0.001 comparison of the blood glucose levels of carveol- and metformin-treated groups vs. diabetic control group.

### Effect on Glucose Tolerance

**Figure 4:**
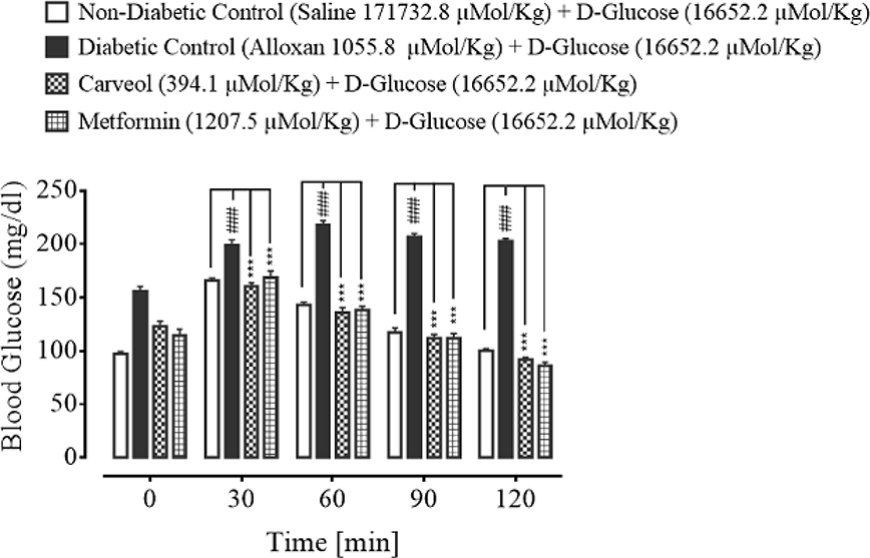
Bar graph representing blood glucose levels at different time intervals (0-120) after administration of oral glucose load (non-diabetic control), alloxan-treated (diabetic control), carveol-treated, and metformin-treated groups. Values expressed as mean ± SEM. One-way ANOVA was used for statistical analysis, followed by post-hoc Tukey’s test. ^###^ *p* < 0.001 vs. saline group and ^***^ *p* < 0.001 comparison of the blood glucose levels of carveol- and metformin-treated groups vs. diabetic control group.

The blood glucose levels of non-diabetic control (saline, 10 mL/Kg), diabetic control alloxan-treated (1055.8 µMol/Kg), carveol-treated (394.1 µMol/Kg) and metformin-treated (positive control) (1207.5 µMol/Kg) groups at the different time intervals (0 – 120 min) are shown in **Figure 4**.

### Effect on HbA1C

The HbA1C levels of non-diabetic control (saline, 10 mL/Kg), diabetic control alloxan-treated, carveol-treated (65.7, 197, and 394.1 µMol/Kg), and metformin-treated (positive control) (1207.5 µMol/Kg) groups are shown in **Table 4**.

**Table 4:** Effect of carveol at different doses and metformin on glycosylated hemoglobin A1C (HbA1C) in alloxan-induced diabetic rats.

**Group**	**HbA1c level (%)**
Non-Diabetic Control (Saline, 10 mL/Kg)	5.0 ± 0.05
Diabetic Control (Alloxan 1055.8 µMol/Kg)	7.1 ± 0.11^###^
Alloxan (1055.8 µMol/Kg) + Carveol (65.7 µMol/Kg)	5.6 ± 0.14^***^
Alloxan (1055.8 µMol/Kg) + Carveol (197 µMol/Kg)	5.2 ± 0.11^***^
Alloxan (1055.8 µMol/Kg) + Carveol (394.1µMol/Kg)	4.9 ± 0.06^***^
Alloxan (1055.8 µMol/Kg) + Metformin (1207.5 µMol/Kg)	5.1 ± 0.10^***^

Values expressed as mean ± SEM. One-way ANOVA was used for statistical analysis, followed by post-hoc Dunnett test. ^###^p < 0.001 vs. saline group, and ^***^p < 0.001 comparison of the HbA1C levels of carveol- and metformin-treated groups vs. diabetic control group.

### Effect on Lipid Profile

The serum levels of TGs, TC, LDL, and HDL of non-diabetic control (saline 10 mL/Kg), diabetic control alloxan-treated, carveol-treated (65.7, 197, and 394.1 µMol/Kg), and metformin-treated (positive control) groups are shown in **Table 5**.

**Table 5:** Effect of carveol on levels of triglycerides (TGs), total cholesterol (TC), low-density lipoprotein (LDL), and high-density lipoprotein (HDL) in alloxan-induced diabetic rats.

Treatment	TGs (mg/dl)	TC (mg/dl)	LDL (mg/dl)	HDL (mg/dl)
Non-Diabetic Control (Saline, 10 mL/Kg)	119.2 ± 2.31	85.2 ± 3.70	67.6 ± 1.86	50.4 ± 1.07
Diabetic Control (Alloxan 1055.8 µMol/Kg)	169.5 ± 3.09^##^	176.2 ± 3.32^###^	96.2 ± 3.11^##^	39.5 ± 1.70^##^
Alloxan (1055.8 µMol/Kg) + Carveol (65.7 µMol/Kg)	131.3 ± 2.84^***^	174.7 ± 9.94	75.2 ± 4.87^*^	50.2 ± 1.65^**^
Alloxan (1055.8 µMol/Kg) + Carveol (197 µMol/Kg)	122.3 ± 14.4^***^	163.6 ± 13.5	73.6 ± 4.17^*^	50.6 ± 4.9^*^
Alloxan (1055.8 µMol/Kg) + Carveol (394.1µMol/Kg)	120.4 ± 6.88^***^	163.2 ± 10.8	65 ± 7.36^***^	51 ± 2.08^**^
Alloxan (1055.8 µMol/Kg) + Metformin (1207.5 µMol/Kg)	128.2 ± 3.49^***^	166 ± 3.36	84.5 ± 2.59	54 ± 1.22^***^

Values expressed as mean ± SEM. One-way ANOVA was used for statistical analysis, followed by post-hoc Dunnett test. ^##^p < 0.01, ^###^p < 0.001 vs. saline group, and ^*^p < 0.05, ^**^p < 0.01, and ^***^p < 0.001 comparison of the blood glucose levels of carveol- and metformin-treated groups vs. diabetic control group.

### Effect on Hepatic Enzymes

The liver enzyme levels AST, ALT, ALP, and TB of non-diabetic control (saline 10 mL/Kg), diabetic control alloxan-treated (1055.8 µMol/Kg), carveol-treated (65.7, 197, and 394.1 µMol/Kg), and metformin-treated (positive control) (1207.5 µMol/Kg) groups are shown in **Table 6**.

**Table 6:** Effect of Carveol on levels of alanine transaminase (ALT), aspartate aminotransferase (AST), alkaline phosphatase (ALP), and total bilirubin (TB) in alloxan-induced diabetic rats.

Treatment	ALT (u/L)	AST (u/L)	ALP (u/L)	TB (mg/dl)
Non-Diabetic Control (Saline, 10 mL/Kg)	31.8 ± 2.15	28.2 ± 1.2	178.8 ± 2.67	0.8 ± 0.07
Diabetic Control (Alloxan 150 mg/Kg)	141.2 ± 8.49^###^	70.5 ± 2.21^###^	345 ± 2.64^###^	1.65 ± 0.15^###^
Alloxan (150 mg/Kg) + Carveol (65.7 µMol/Kg)	38 ± 2.04^***^	27.7 ± 3.19^***^	200.2 ± 10.18^***^	0.67 ± 0.07^***^
Alloxan (150 mg/Kg) + Carveol (197 µMol/Kg)	28.6 ± 6.96^***^	25 ± 3.46^***^	185.3 ± 13.54^***^	0.76 ± 0.08^***^
Alloxan (150 mg/Kg) + Carveol (394.1 µMol/Kg)	25.6 ± 3.12^***^	23.8 ± 2.72^***^	186.8 ± 5.57^***^	0.7 ± 0.1^***^
Alloxan (150 mg/Kg) + Metformin (200 mg/Kg)	32.2 ± 3.63 ^***^	33 ± 1.29^***^	200 ± 4.26^***^	0.85 ± 0.06 ^***^

Values expressed as mean ± SEM. One-way ANOVA was used for statistical analysis, followed by post-hoc Dunnett test. ^###^p < 0.001 vs. saline group, and ^***^p < 0.001 comparison of the blood glucose levels of carveol- and metformin-treated groups vs. diabetic control group.

## Discussion

Virtual screening, or the *in-silico* approach, is a procedure through which ligands are docked with respective target proteins by using a fast and cost-effective technique, and it requires computer-assisted programs and software (Prakhov et al., 2010). We demonstrated a comparative study by comparing the results for ligand–protein complexes to those for the standard drugs, obtained from the RCSB and PubChem database. Auto-Dock vina, patch dock, gromacs, and gold suite provide docking of ligands with a possibility of a dozen torsional degrees of freedom. A lower binding value (kcal/mol) reveals reduced energy of desolvation, which depicts stability of the ligand–protein complex (Pecsi et al., 2010). Hydrogen bonding, pi–pi bonding, and other hydrophobic interactions provide valuable strength for structurally complex stabilization. Other hydrophobic interactions play a vital role in increasing the affinity of the ligand towards the protein receptor (Dallakyan and Olson, 2015). E-value, H bonding, and hydrophobic interactions play a key role in the assessment of the binding affinity of ligand and protein complexes. We demonstrated here that carveol manifested the best binding score with the lowest E value against SGLT. Based on the E value against different protein targets, the order of ligand affinity was found to be SGLT > AR > SIMI > PI3K > 11β-HSD1 > FBP1 > C-AG > PEPCK > GSK-3β > AA > PPAR-γ.

The enzyme alpha-amylase and alpha-glucosidase are responsible for the breakdown of carbohydrates and for hydrolyzing starch into glucose before absorption (Chelladurai and Chinnachamy, 2018). Reduction in postprandial hyperglycemic level is due to alpha-amylase inhibition, which delays the small intestine carbohydrate digestion and diminishes the postprandial blood glucose level (Kwon et al., 2007). One of the approaches for treating DM is to inhibit the carbohydrate digesting enzymes such as * α*-amylase in the GIT and thereby reduce postprandial glucose (Raman et al., 2012). Previous studies demonstrated that flavonoids, tannins, and terpenoids could effectively inhibit alpha-amylase and alpha-glucosidase (Khan et al., 2014). Similarly, we demonstrated that carveol can inhibit α-amylase concentration-dependently, which may be due to its mono-terpenoid nature. Moreover, α-amylase inhibition was performed at various concentrations and compared with the standard drug acarbose at the same concentrations.

In the present study, the antidiabetic effect of carveol against alloxan-induced diabetes in rats was investigated. Administration of alloxan leads to inhibition of insulin secretion, resulting in persistent hyperglycemia or diabetes (Yang et al., 2010). Carveol reversed the blood glucose level in a dose-dependent and time-dependent fashion when compared to the alloxan-treated diabetic group. Furthermore, the results for carveol were comparable to those for the standard metformin group, which was used as a positive control. Metformin lowers the blood glucose level by numerous pathways, including decreasing biogenesis in hepatic tissue, minimizing glucose absorption by the intestine, and improving its peripheral utilization through enhancing insulin sensitivity (Klip and Leiter, 1990). We demonstrated that carveol reduced the body weights of test animals at regular daily intervals. As obesity is directly related to diabetes, a drug with the dual benefits of glycemic and weight control is of interest in diabetes, so this is an attractive outcome of carveol usage. In the glucose-loaded hyperglycemia model, which aims to assess oral glucose tolerance, carveol exhibited considerably better tolerance of glucose overload at regular intervals than did the metformin group. Carveol produced a dose-dependent effect of reducing the HbA1C level and was found to be effective as a long-term antidiabetic agent.

Diabetes is also associated with a variable lipid profile (Virdi et al., 2003), which can be further linked to obesity and renal impairment (Al-Shamaony et al., 1994). The marked hyperlipidemia that characterizes the diabetic state may be a consequence of the abnormal function of lipolytic hormones on the fat depots (Ayeleso et al., 2012). Lowering of serum lipid levels through dietary or drug therapy seems to be associated with a decrease in the risk of vascular disease in diabetes (Ayeleso et al., 2012). The results of the present investigation show that carveol produced a significant decrease in TC, TG, and LDL and a significant increase in HDL as compared to diabetic control. This improvement in the lipid profile status of alloxan-treated rats revealed the anti-hyperlipidemic properties of carveol. It has previously been reported that about 30% of blood cholesterol is circulated in the form of HDL-cholesterol (HDL-C). HDL-C removes cholesterol atheroma from arteries and transports it to the liver for excretion or re-utilization, which is a beneficial approach in cardiovascular diseases (Owolabi et al., 2010). Therefore, the increase in the serum HDL-C level by carveol in hyperglycemic rats indicates that carveol can augment HDL-C effects. Furthermore, our study demonstrated that various hepatic enzymes, such as AST, ALT, and ALP, were significantly increased in the alloxan-induced diabetic control group. However, carveol significantly decreased the level of these enzymes compared to diabetic control. Furthermore, increased gluconeogenesis and ketogenesis might be due to elevated activity of transaminases (Gandhi et al., 2011) associated with hepatocyte damage in experimental animals (Abolfathi et al., 2012). The ability of carveol to attenuate the serum level of ALT, AST, and ALP suggests its hepato-cellular protective effect.

## Conclusions

The present study demonstrated that carveol exhibited maximum binding affinity against SGLT, intermediate binding affinity against FBP1, and lower energy values against PEPCK and GSK-3β. Moreover, our *in-vitro* and *in-vivo* study suggests that carveol could be a promising therapeutic agent in the management of diabetes. However, extensive exploration is still required to delineate the underlying protective mechanisms of carveol.

## Data Availability Statement

All datasets generated and analyzed for this study are included in the article/**Supplementary Material**.

## Ethics Statement

The animal study was reviewed and approved by Ethical Committee, Riphah Institute of Pharmaceutical Sciences (Ref. No.: REC/RIPS/2019/03).

## Author Contributions

MA carried out the computational studies, in-vitro and in-vivo experimentation, evaluation of results, and documentation. A-uK, and FS supervised the research project and drafted the initial and final version of the manuscript. LK help in data analysis, revision and with drug Carveol. All authors contributed to the article and approved the submitted version.

## Conflict of Interest

The authors declare that the research was conducted in the absence of any commercial or financial relationships that could be construed as a potential conflict of interest.
