# Supplementary figures and images for: Computational and Pharmacological Evaluation of Carveol for Antidiabetic Potential
Source: Front Pharmacol. 2020 Jul 29;11:919. doi: 10.3389/fphar.2020.00919 (PMC7403477; doi:10.3389/fphar.2020.00919)

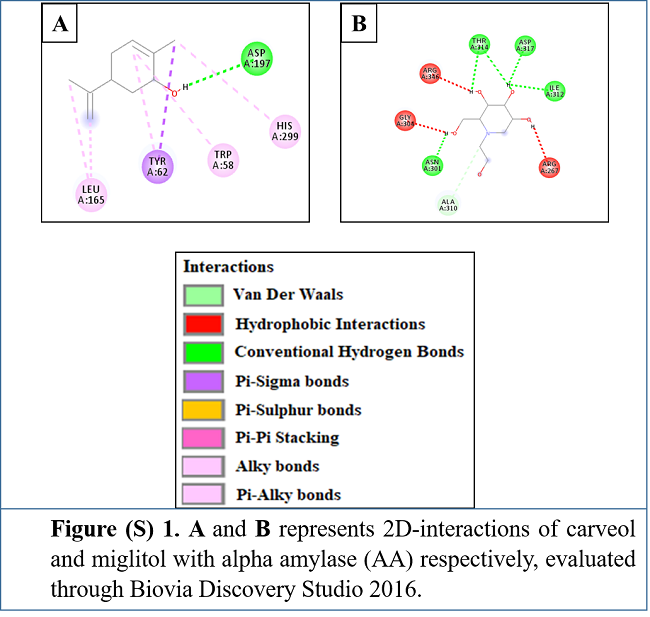

Supplement: Supplementary file 1 [file Image_1.tif]

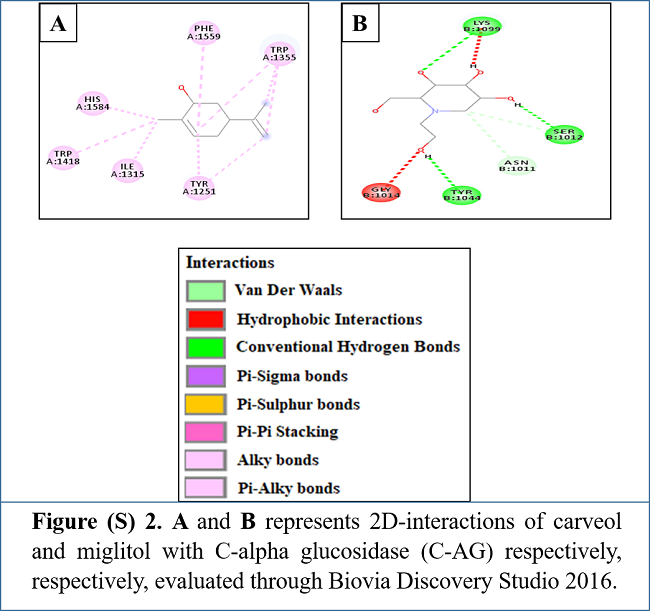

Supplement: Supplementary file 2 [file Image_2.tif]

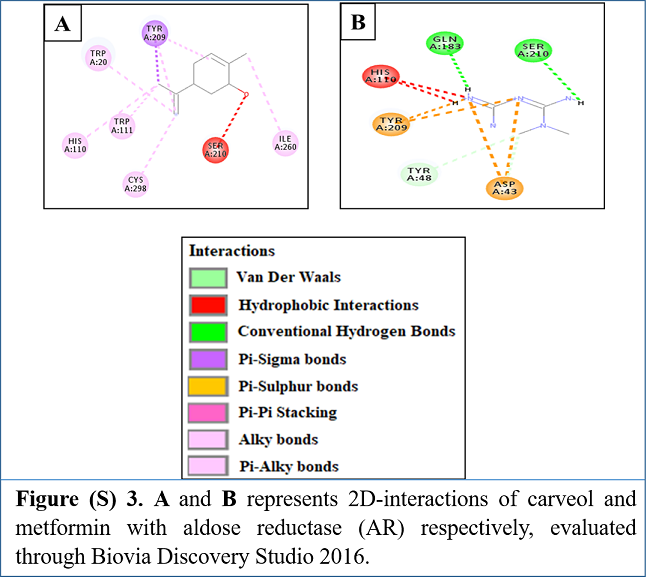

Supplement: Supplementary file 3 [file Image_3.tif]

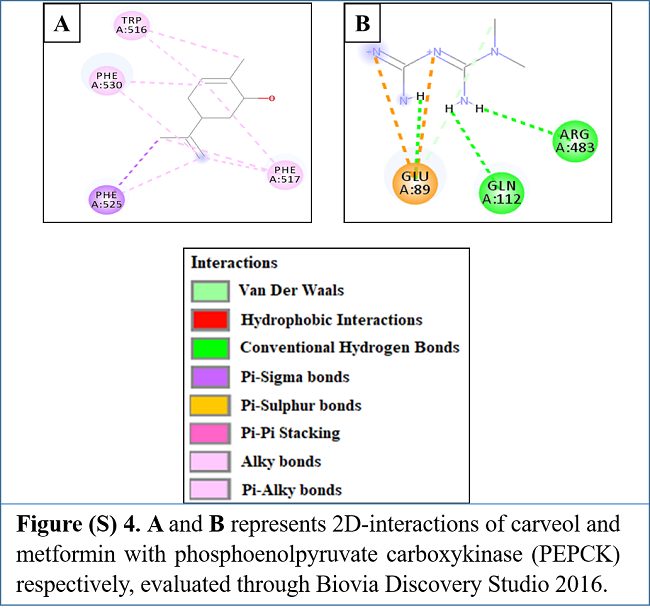

Supplement: Supplementary file 4 [file Image_4.tif]

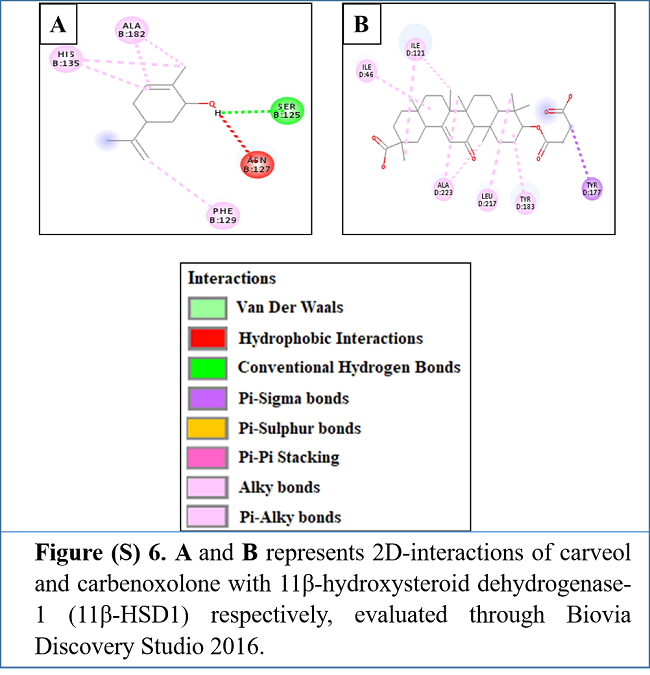

Supplement: Supplementary file 5 [file Image_6.tif]

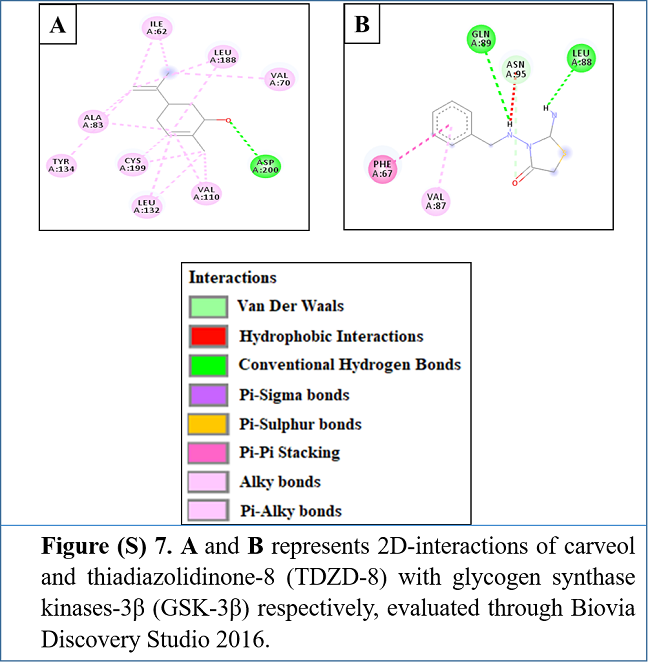

Supplement: Supplementary file 6 [file Image_7.tif]

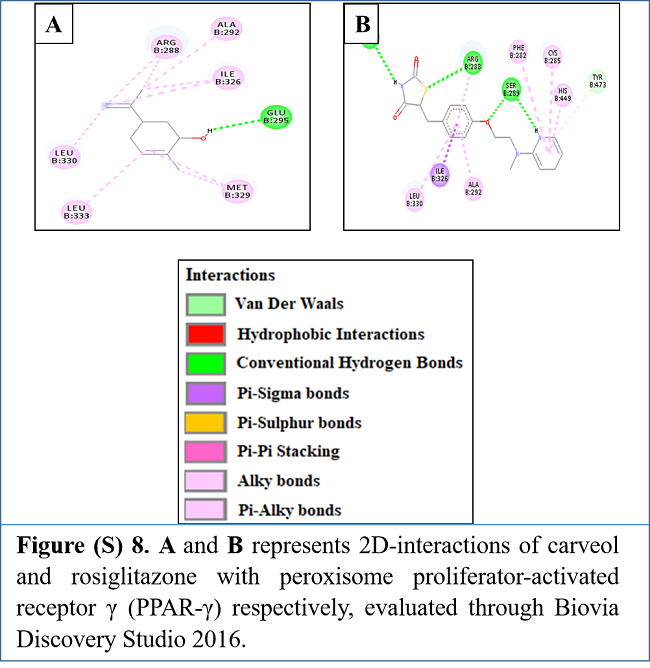

Supplement: Supplementary file 7 [file Image_8.tif]

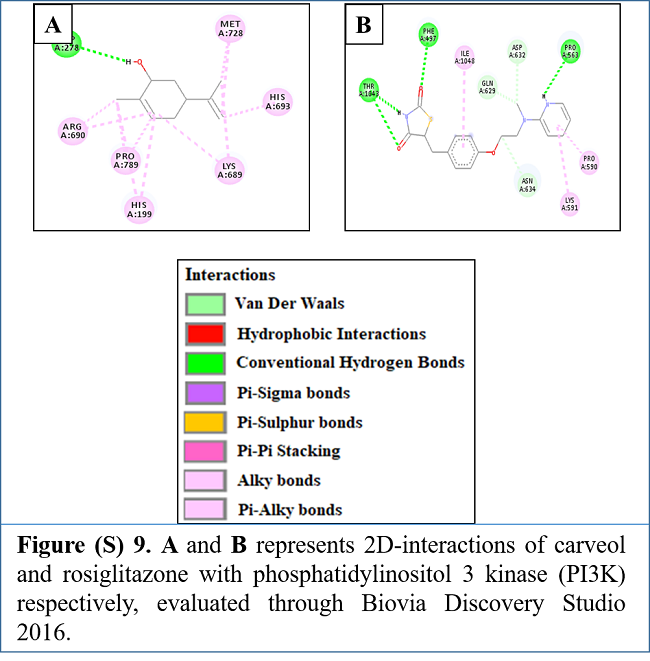

Supplement: Supplementary file 8 [file Image_9.tif]

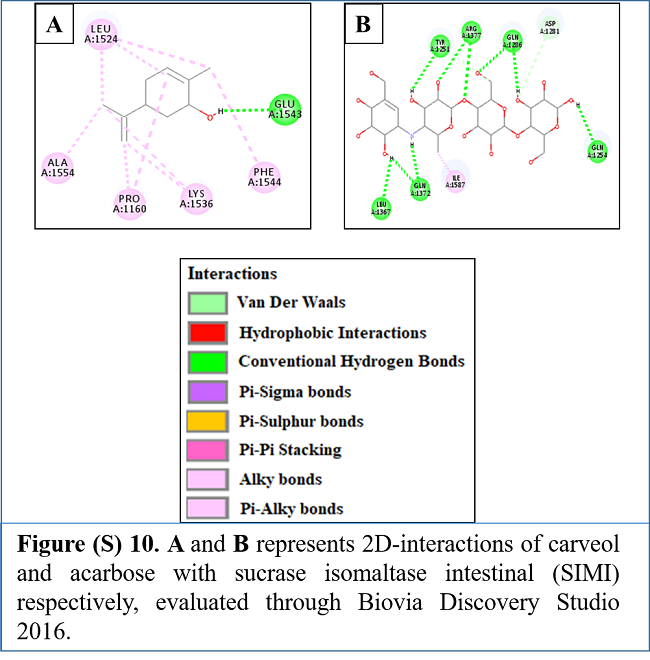

Supplement: Supplementary file 9 [file Image_10.tif]
